# Impact of Teriparatide and Denosumab on Clinical and Radiographic Outcomes in Osteoporotic Vertebral Compression Fractures

**DOI:** 10.3390/medicina60081314

**Published:** 2024-08-14

**Authors:** Byung-Taek Kwon, Dae-Woong Ham, Sang-Min Park, Ho-Joong Kim, Jin S. Yeom

**Affiliations:** 1 Department of Orthopedics, Chung-Ang University College of Medicine, Chung-Ang University Gwangmyeong Hospital, Gwangmyeong 14353, Republic of Korea; osbtkwon@gmail.com; 2 Department of Orthopedics, Chung-Ang University College of Medicine, Chung-Ang University Hospital, Seoul 06973, Republic of Korea; hamdgogo@gmail.com; 3 Spine Center and Department of Orthopaedic Surgery, Seoul National University College of Medicine, Seoul National University Bundang Hospital, Seongnam-si 13620, Republic of Korea; oshjkim@gmail.com (H.-J.K.); highcervical@gmail.com (J.S.Y.)

**Keywords:** bone mineral density, denosumab, osteoporosis, teriparatide, vertebral compression fracture

## Abstract

*Background and Objectives:* Osteoporotic vertebral compression fractures (OVCFs) are prevalent among the elderly, often leading to significant pain, morbidity, and mortality. Effective management of underlying osteoporosis is essential to prevent subsequent fractures. This study aimed to compare the clinical and radiographic outcomes of teriparatide and denosumab treatments in patients with OVCFs to determine their relative effectiveness in improving patient outcomes. *Materials and Methods:* This retrospective study included 78 patients diagnosed with an acute thoracolumbar OVCF who received either teriparatide (35 patients) or denosumab (43 patients) within three months of a fracture. Clinical outcomes were assessed using the visual analog scale (VAS) for back pain, Oswestry disability index (ODI), and EQ-5D quality of life scores at baseline, 6 months, and 12 months. Bone mineral density (BMD) and radiographic outcomes were evaluated initially and at 12 months post-treatment. *Results:* Both treatment groups demonstrated significant improvements in VAS, ODI, and EQ-5D scores over 12 months. No significant differences were observed between the teriparatide and denosumab groups in terms of clinical outcomes or radiographic measurements at any time point. Fracture union and BMD improvements were similarly observed in both groups. The teriparatide group had a lower baseline BMD, but this did not affect the overall outcomes. *Conclusions:* Both teriparatide and denosumab are effective in improving clinical and radiographic outcomes in patients with OVCFs. Despite concerns about denosumab’s potential to hinder fracture healing, our study found no significant differences between the two treatments. These findings support the use of denosumab for early treatment of OVCFs to prevent subsequent fractures without compromising fracture healing. Further prospective studies are needed to confirm these results.

## 1. Introduction

Osteoporotic vertebral compression fractures (OVCFs) are common in the elderly and are associated with significant back pain, morbidity, and mortality [1,2,3,4,5]. Effective treatment is needed to relieve symptoms and prevent complications [6,7,8]. Managing the underlying osteoporosis is crucial due to the high risk of additional fractures [9]. Conservative treatments, such as bed rest, pain medication, a brace, and some kinds of interventions, are necessary for acute OVCF management [9]. Osteoporosis treatment following OVCF is important to reduce the risk of subsequent OVCFs [10].

Osteoporosis medications enhance bone strength and decrease fracture risk in osteoporotic patients [1]. Bisphosphonates and denosumab are commonly used medications for osteoporosis management [11]. In cases of severe fractures or severe osteoporosis, teriparatide is sometimes used [12]. Bisphosphonates are widely used for the treatment of osteoporosis and have been shown to reduce bone turnover and improve bone mineral density (BMD). They are considered first-line medicines for the prevention of fractures and repeat fractures in patients with osteoporosis [13]. Denosumab, an inhibitor of receptor activator of nuclear factors κB ligand (RANKL), is another medication commonly used in the management of osteoporosis. It has been shown to be effective in treating postmenopausal osteoporosis and reducing fracture risk [14]. Studies suggest denosumab may be more effective than bisphosphonates in lowering vertebral fracture risk [15]. Teriparatide, a parathyroid hormone fragment, has shown promise in treating OVCFs by enhancing BMD, fracture healing, and improving pain intensity and functional recovery [11,12,16]. Teriparatide has been linked to better functional outcomes, reduced pain, and improved physical health in patients with multiple OVCFs [17].

Despite their different mechanisms, both medications aim to reduce fracture risk. Teriparatide is often preferred for severe osteoporosis or multiple fractures due to its bone formation-stimulating effects, which may accelerate fracture healing. Denosumab decreases bone resorption and turnover, but does not stimulate fracture healing, though further research is needed to clarify its impact. This study compares the outcomes of teriparatide and denosumab treatments in OVCF patients to optimize osteoporosis treatment strategies after an OVCF.

## 2. Material and Methods

### 2.1. Study Design and Patient Selection

This study is a retrospective study to evaluate the clinical and radiographic outcomes of a conservative treatment for thoracolumbar OVCF conducted at a tertiary educational hospital from March 2017 to March 2020. This study was approved by the institutional review board at our hospital (B-2101-661-108) and conducted in accordance with the Declaration of Helsinki. Informed consent was waived due to the retrospective nature of this study. The data were collected from the electronic medical records and included patients’ demographic information, clinical and radiographic data, and surgical details.

Patients included in this study were previously diagnosed with one or more acute OVCFs. The fractures were confirmed by high signal intensity on fat-suppressed T2-weighted images using magnetic resonance imaging. Only the patients who underwent conservative treatment and received either teriparatide or denosumab within three months after fracture were considered for inclusion. All patients were instructed to wear a thoraco-lumbar-sacral orthosis (TLSO) brace for three months from the time of the fracture diagnosis.

**Figure 1:**
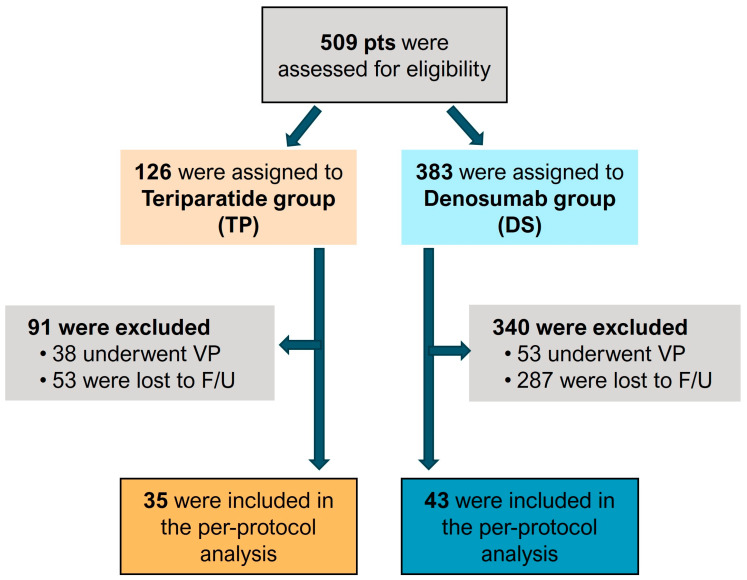
Flow diagram of this study. Abbreviations: VP—vertebroplasty; F/U—follow-up.

The exclusion criteria were defined to ensure the accuracy and relevance of the study’s results. Patients who had undergone any invasive procedures, such as vertebroplasty or kyphoplasty, were excluded. Furthermore, those with a history of a pedicle screw fixation were not included in the study. The study also excluded patients with neoplastic malignancies, infections, or any contraindications to teriparatide or denosumab treatment. Patients receiving ongoing treatment with osteoporotic medication, such as bisphosphonates, denosumab, teriparatide, and hormone replacement therapy, and/or steroid treatment were excluded due to their medical histories. And we also excluded patients diagnosed with ankylosing spondylitis. Additionally, patients who discontinued teriparatide or denosumab treatment prematurely, who received treatment for less than one year, or who received crossover therapy were excluded from this study. After applying the exclusion criteria, 78 patients were included in the final analysis (Figure 1).

### 2.2. Clinical Outcomes

Patient demographics, fracture details, treatment details, and bone density measurements were systematically collected. The specific variables included demographics such as age, sex, height, weight, and BMI. For fracture details, the segments and the number of fractured vertebral bodies were collected. Bone mineral density (BMD) was measured using dual energy X-ray absorptiometry (DEXA, Hologic Inc., Waltham, MA, USA). DEXA was used to measure BMD initially and at one year post-treatment (T-score and BMD [g/cm^2^]) for the spine, femur neck, and hip. When the T-score was lower than −2.5, the patient was counted as osteoporotic. The choice of osteoporosis medication was determined as follows: teriparatide treatment was administered to patients with a T-score of −3.0 or below who consented to the treatment and could continue teriparatide for more than one year, provided there were no contraindications based on their general condition. For all other patients, denosumab or bisphosphonate treatment was administered. All patients were also administered calcium and vitamin D supplements.

Patient-reported outcome measures (PROMs) were evaluated at three time points: initial, and at 6 and 12 months after injury. Clinical assessments included the visual analog scale (VAS) for low back pain, the Oswestry disability index (ODI) [18], and the European quality of life-5 dimensions (EQ-5D) score [19]. The VAS pain score was used to measure the intensity of low back pain, ranging from 0 (no pain) to 100 (severe pain), with higher scores indicating greater pain. The ODI score assessed disability levels in daily activities due to low back pain, providing a percentage score where higher values reflect greater disability. This tool is widely used for evaluating the functional status of patients with spinal disorders. The EQ-5D value evaluated the quality of life (QOL) across five dimensions: mobility, self-care, usual activities, pain/discomfort, and anxiety/depression. The EQ-5D value ranged from −0.066 to 1.000, with 1 representing the best possible quality of life.

### 2.3. Radiographic Assessment

All patients underwent evaluation with plain radiographs, CT, or MRI. Initial and final follow-up radiographs were used to confirm fractures, and to measure vertebral body height and vertebral wedge angle. Radiographs were analyzed using a picture archiving and communication system (INFINITT Healthcare, Seoul, Republic of Korea) [20]. The heights of the anterior (A) columns of the fractured vertebral bodies were measured to determine the compression rate (CR). To correct for magnification bias, the mean height of the superior (R1) and inferior (R2) adjacent vertebral bodies was used as a reference (R = [R1 + R2]/2). CR were calculated as CR = (1 − (A)/R) × 100. The wedge angle of the fractured vertebral body was measured as the angle between the superior and inferior endplates. Dynamic radiographs (e.g., translateral radiographs taken in prone and supine positions) were used to confirm bone union. Bone union was confirmed by comparing dynamic radiographs with standing radiographs; if there was no motion in the vertebral body, bone union was considered to be achieved [21]. All parameters were expressed as percentages. Radiographic measurements were evaluated by two orthopedic surgeons and presented as mean values.

### 2.4. Statistical Analysis

Descriptive statistics were employed to summarize the demographic and clinical characteristics of the study population. The normality of continuous variables was assessed using the Shapiro–Wilk test. Continuous variables were presented as means and standard deviations for normally distributed data, and as medians and interquartile ranges (IQR) for non-normally distributed data. Categorical variables were expressed as frequencies and percentages. Statistical comparisons between the two groups were performed to identify significant differences in baseline characteristics and outcomes. Independent t-tests were used for continuous variables, and chi-square tests were applied for categorical variables. For continuous variables that did not pass the normality test, the Mann–Whitney U test was employed to compare medians and IQRs between the groups using non-parametric methods. To evaluate the impact of the osteoporosis medication on clinical outcomes, changes in these outcomes over time were analyzed. Generalized estimating equations (GEE) with an exchangeable working correlation structure and Gaussian family were used to account for repeated measures within individuals and to analyze the effects of time and age group on clinical outcomes. The GEE models included the main effects of the time and treatment group, as well as their interaction terms, to determine whether changes in the clinical outcomes over time differed by treatment group. Statistical analysis was conducted using Stata/MP 17.1 (StataCorp LLC, College Station, TX, USA), with a significance level set at *p* < 0.05.

## 3. Results

### 3.1. Patient Characteristics

A total of 509 patients were assessed for eligibility. After applying the exclusion criteria, our study included 78 patients, with 35 in the teriparatide group and 43 in the denosumab group. The mean age was 77.6 years in the teriparatide group and 76.2 years in the denosumab group, with no significant difference (*p* = 0.32). The gender distribution showed a significant difference (*p* = 0.033), with the teriparatide group having 6% men and 94% women, while the denosumab group had 23% men and 77% women. The mean BMI was similar between the groups (23.4 for teriparatide and 23.8 for denosumab, *p* = 0.65).

Fracture segments were distributed similarly across both groups, with the majority of patients having one or two fractured vertebrae. There was no significant difference in the extent of segments between the groups (*p* = 0.32). The locations of fractures were also comparable, predominantly affecting the lumbar region (*p* = 0.44). Initial DEXA measurements showed that the teriparatide group had significantly lower BMD values compared to the denosumab group (Table 1).

### 3.2. BMD and Radiographic Outcomes

At the 12-month follow-up, the teriparatide group continued to show lower BMD values compared to the denosumab group. The absolute change in BMD values from initial measurements to those observed 12 months after the fracture showed no significant difference between the two groups. Similarly, the relative changes in BMD, calculated as a percentage of the initial values, showed no significant differences between the two groups.

Initial compression rates and wedge angles were comparable between the groups, with no significant differences observed. The final compression rates and wedge angles at the 12-month follow-up also showed no significant differences between the groups. Bone union rates were 74% for the teriparatide group and 81% for the denosumab group, with no significant difference (*p* = 0.45). In the teriparatide group, three patients (9%), and in the denosumab group, four patients (9%) experienced new vertebral fractures apart from the index vertebra, with no statistically significant difference (*p* > 0.99) (Table 2).

### 3.3. Clinical Outcomes

The VAS pain scores for the back showed a significant overall time effect (*p* = 0.011) but no significant group effect (*p* = 0.755). Similarly, the ODI score demonstrated a significant overall time effect (*p* < 0.001) but no significant group effect (*p* = 0.056). The EQ-5D scores improved significantly over time (*p* = 0.001), with no significant group effect (*p* = 0.259). Both treatment groups showed improvements in clinical outcomes over time, but there was no significant difference between the two medications (teriparatide and denosumab) in terms of their effects on these outcomes. While the initial clinical outcomes might have been worse for the teriparatide group, the differences in outcomes between the two groups at any specific time point were not statistically significant (all *p*-values > 0.05) (Table 3). Furthermore, there were no complications related to the treatments, including osteonecrosis of the jaw, atypical fractures, and hypercalcemia, in either group.

## 4. Discussion

This study aimed to compare the therapeutic effects of teriparatide and denosumab in patients with OVCFs. In this study, significant improvements in back pain, functional outcomes, and quality of life were observed in both the teriparatide and denosumab groups over a 12-month period. Furthermore, fracture healing and BMD improvement in both treatment groups were also similarly observed. These improvements were consistent regardless of the treatment applied, indicating that both osteoporosis treatments effectively contribute to functional recovery in patients with OVCFs.

Osteoporosis management is crucial, particularly for preventing subsequent fractures following an initial OVCF [9,22]. Anti-resorptive agents, such as bisphosphonate and denosumab, are commonly used; however, their ability to decrease bone turnover has raised concerns about their potential to hinder fracture healing. Although some evidence supports denosumab’s efficacy in suppressing osteoclast activity and reducing bone pain in cases of bone metastasis, its impact on osteoporotic fracture healing remains under-researched [23]. Animal studies have shown that denosumab increases callus volume and delays remodeling during fracture healing, yet these effects do not appear to impede the overall healing process or functional recovery in clinical settings [24].

Teriparatide is well documented for its role in promoting fracture healing, with numerous studies supporting its effectiveness in enhancing bone union and improving clinical outcomes in vertebral fractures in patients with OVCFs [12]. The mechanisms underlying the observed pain reduction and functional recovery in patients treated with teriparatide may be attributed to its anabolic action, which enhances fracture healing [25]. Teriparatide’s potential to inhibit inflammatory cytokines such as IL-1β, IL-6, and TNF-α may also contribute to its pain-relieving effects [26]. These findings align with previous studies that have reported the effectiveness of teriparatide in alleviating pain and preventing vertebral collapse in osteoporotic vertebral fractures.

Radiographically, teriparatide has been shown to be more effective in inhibiting the progression of vertebral deformity compared to bisphosphonates [27]. This study corroborates these findings, demonstrating less vertebral collapse and kyphotic change in patients treated with teriparatide. While denosumab, another anti-resorptive agent, showed similar radiological outcomes to teriparatide [11,12], it differs from bisphosphonates in that it enhances both the bone mineral content and density, as well as mechanical properties, thus not impairing bone formation or healing [24]. Our study confirmed these findings, showing no significant differences in clinical outcomes or bone union rates between the two groups, although the teriparatide group had a lower baseline BMD, which could have influenced union rates.

An important consideration in clinical practice is determining the appropriate timing to begin administering anti-resorptive medications. There are concerns that these agents may interfere with the healing process of fractures, which could lead to prolonged disabilities and delayed recovery of function. However, our study suggests that denosumab does not adversely affect clinical or radiographic outcomes compared to teriparatide. Furthermore, the new vertebral fracture rate following OVCF is generally reported to be around 20–35% [28,29,30]. In the present study, the new vertebral fracture rate was relatively lower in both groups, at 9% (3/35) in the teriparatide group and 9% (4/43) in the denosumab group. Although the baseline BMD was significantly lower in the teriparatide group, the observed subsequent fracture rates were lower than those typically reported. This finding supports the early use of denosumab to prevent subsequent fractures, without the risk of hindering bone healing.

This study has several limitations. Firstly, the retrospective design may introduce selection bias and limit the ability to establish causality. The decision to treat patients with either teriparatide or denosumab was influenced by clinical judgment, patient history, and individual preferences, which could introduce a selection bias towards certain patient characteristics or clinical conditions. Consequently, patients in the teriparatide group showed statistically lower initial BMD compared to those in the denosumab group. This difference should be considered when interpreting the results of the present study. Secondly, the relatively small sample size may not capture all potential differences between the treatment groups. This limitation underscores the need for larger, multi-center studies to validate the findings and enhance their generalizability. Additionally, this study did not account for certain patient-specific factors that could influence treatment outcomes, such as baseline physical activity levels, dietary habits, and adherence to concomitant osteoporosis medications or supplements. These factors can significantly impact bone health and fracture healing but were not systematically controlled or reported in this study. While this study offers important preliminary insights into the comparative efficacy of teriparatide and denosumab in managing OVCFs, the limitations highlight the need for more robust, prospective research. Future studies should employ randomized controlled designs with larger sample sizes, comprehensive follow-up assessments, and advanced imaging techniques to validate and extend these findings. Additionally, accounting for a broader range of patient-specific factors and expanding the study population to include diverse groups will enhance the applicability and relevance of the research outcomes.

## 5. Conclusions

In this study, both teriparatide and denosumab were effective in improving clinical outcomes in patients with OVCFs. Despite concerns regarding denosumab’s impact on fracture healing, our study found no significant differences in clinical or radiographic outcomes compared to teriparatide. These findings suggest that denosumab can be safely used in the early treatment of OVCFs to prevent subsequent fractures without compromising fracture healing.

## Figures and Tables

**Table 1:** Patient characteristics and clinical manifestations.

Characteristics	Teriparatide (*n* = 35)	Denosumab (*n* = 43)	*p*-Value
Age	77.6 (6.1)	76.2 (6.5)	0.32
Sex (M:F), No. (%)	2 (6%):33 (94%)	10 (23%):33 (77%)	0.033
BMI	23.4 (3.3)	23.8 (3.4)	0.65
Extent of levels, No. (%)			0.32
1	25 (71%)	34 (79%)	
2	7 (20%)	6 (14%)	
3	0 (0%)	2 (5%)	
>3	3 (9%)	1 (2%)	
Locations, No. (%)			0.44
L	18 (51%)	27 (63%)	
T	13 (37%)	13 (32%)	
T and L	4 (12%)	3 (7%)	
DEXA			
Lumbar (T-score)	−3.7 (1.1)	−2.5 (0.9)	<0.001
BMD of lumbar (g/cm^2^)	0.722 (0.144)	0.854 (0.134)	<0.001
BMD of femur neck (g/cm^2^)	0.503 (0.114)	0.610 (0.109)	<0.001
BMD of total hip (g/cm^2^)	0.609 (0.108)	0.741 (0.108)	<0.001
Medical comorbidity, No. (%)			
Diabetes	6 (17%)	7 (16.3%)	>0.99
Hypertension	16 (46%)	14 (33%)	0.34
Cerebrovascular disease	6 (17%)	4 (9%)	0.49
Chronic kidney disease	1 (3%)	4 (9%)	0.49

M—male; F—female; BMI—body mass index; L—lumbar; T—thoracic; DEXA—dual-energy X-ray absorptiometry; BMD—bone mineral density. Data are presented as mean (standard deviation), unless otherwise indicated.

**Table 2:** Outcomes during follow-up between groups.

Characteristics	Teriparatide (*n* = 35)	Denosumab (*n* = 43)	*p*-Value
DEXA at 12-month			
Lumbar (T-score)	−3.5 (1.0)	−2.2 (0.9)	<0.001
BMD of lumbar (g/cm^2^)	0.776 [0.708–0.881]	0.915 [0.841–0.974]	<0.001
BMD of femur neck (g/cm^2^)	0.500 (0.101)	0.628 (0.115)	<0.001
BMD of total hip (g/cm^2^)	0.613 (0.098)	0.753 (0.113)	<0.001
DEXA, absolute change *			
Lumbar (T-score)	0.3 (0.5)	0.3 (0.3)	0.86
BMD of lumbar (g/cm^2^)	0.065 (0.191)	0.061 (0.075)	0.91
BMD of femur neck (g/cm^2^)	−0.001 (0.046)	0.014 (0.065)	0.28
BMD of total hip (g/cm^2^)	0.009 (0.043)	0.013 (0.035)	0.66
DEXA, relative change (%) ^†^			
Lumbar (T-score)	6.8(13.8)	14.6(21.3)	0.088
BMD of lumbar	11.3 (21.6)	7.9 (8.8)	0.38
BMD of femur neck	0.3 (9.0)	2.8 (10.5)	0.32
BMD of total hip	1.8 (8.2)	1.8 (4.8)	0.96
Radiographics			
Compression rate (initial, %)	22.8 [15.0–27.4]	21.6 [10.4–35.6]	0.94
Compression rate (final, %)	34.1 [28.8–47.1]	38.5 [33.9–46.0]	0.38
Wedge angle (initial, degree)	7.0 (3.9)	7.7 (3.9)	0.44
Wedge angle (final, degree)	9.6 (5.5)	9.9 (4.6)	0.82
Union, No. (%)	26 (74%)	35 (81%)	0.45
Newly onset vertebral fracture, No. (%)	3 (9%)	4 (9%)	>0.99

M—male; F—female; BMI—body mass index; L—lumbar; T—thoracic; DEXA—dual-energy X-ray absorptiometry; BMD—bone mineral density. Data are presented as mean (standard deviation), unless otherwise indicated. * Difference in the results of DEXA between initial and 12 months after fracture. This is calculated by subtracting the initial BMD value from the 1-year BMD value. ^†^ Relative change in BMD as a proportion of the initial value. It is calculated by dividing the 12-month BMD value by the initial BMD value.

**Table 3:** Clinical outcomes for both groups after fracture during the 12-month follow-up.

Variables	Teriparatide (*n* = 35)	Denosumab (*n* = 43)	*p*-Value
VAS back pain			
Initial	52.7 (16.6)	49.0 (16.4)	
6 months	37.7 (23.1)	37.0 (18.5)	
12 months	38.7 (22.8)	38.1 (17.8)	0.011 ^†^
Overall group effect *	NA	NA	0.755
ODI			
Initial	69.7 (26.7)	63.5 (23.1)	
6 months	30.3 (23.1)	31.4 (24.3)	
12 months	32.6 (27.3)	24.2 (19.8)	<0.001 ^†^
Overall group effect *	NA	NA	0.056
EQ-5D			
Initial	0.444 (0.191)	0.497 (0.219)	
6 months	0.669 (0.249)	0.665 (0.202)	
12 months	0.647 (0.257)	0.656 (0.230)	0.001 ^†^
Overall group effect *	NA	NA	0.259

VAS—visual analog scale; NA—not available; ODI—Oswestry disability index; EQ-5D—European quality of life-5 dimensions. Data are presented as the mean (standard deviation). * *p*-value is from generalized estimating equations for repeated measures comparing treatments during the 12-month follow-up period. ^†^ Overall time effect.

## Data Availability

Data can be obtained from the corresponding author upon reasonable request.
